# Evaluation of Three Imaging Methods to Quantify Key Events in Pelvic Bone Metastasis

**DOI:** 10.3390/cancers16010214

**Published:** 2024-01-02

**Authors:** Haejun Lee, Tae Ran Ahn, Kyung Hoon Hwang, Sheen-Woo Lee

**Affiliations:** 1 Department of Nuclear Medicine, Gachon University Gil Hospital, Incheon 21565, Republic of Korea; cefiro@gilhospital.com (H.L.); khhwang@gilhospital.com (K.H.H.); 2 Department of Radiology, Gachon University Gil Hospital, Incheon 21565, Republic of Korea; ahntr@gilhospital.com; 3 Department of Radiology, The Catholic University of Korea Eunpyeong St. Mary’s Hospital, Seoul 03312, Republic of Korea

**Keywords:** bone metastasis, red marrow hyperplasia, PET/CT, diffusion-weighted MRI, BLADE, RESOLVE

## Abstract

**Simple Summary:**

Accurate assessment of the presence and progression of bone metastases will guide individual treatment plans. Sophisticated cross-sectional imaging techniques, such as magnetic resonance diffusion-weighted imaging (DWI), have gained favor due to their superior soft tissue contrast and resolution without the need for ionizing radiation. Many DWIS are available on clinical scanners, which show promise for objective assessment of bone metastases.

**Abstract:**

Background: The purpose of this study is to compare turbo spin echo diffusion-weighted images in radial trajectory (BLADE DWI) with multi-shot echoplanar imaging (RESOLVE DWI) for imaging the metastatic lesion in the pelvic bone to find a correlation between ADC values and standardized uptake values (SUVs) of FDG uptake in PET/CT. The study also seeks to compare the values of metastatic lesions with those of benign bone lesions, specifically red marrow hyperplasia. Methods: The retrospective IRB-approved study included patients with bone metastasis and red marrow hyperplasia in the pelvic bone who underwent 3.0 T MRI with BLADE/RESOLVE DWI sequences and F-18 FDG PET/CT within one month. BVC (best value comparator) was used in determining the nature of bone lesions. Apparent diffusion coefficient (ADC) and standardized uptake value (SUV) were measured by a radiologist and a nuclear medicine physician. MRI image quality was graded with a Likert scale regarding the visualization of the sacroiliac joint, sacral neural foramen, hamstring tendon at ischial tuberosity, and tumor border. Signal-to-noise ratio (SNR) and imaging time were compared between the two DWIs. Mean, peak, and maximum SUVs between metastatic and benign red marrow lesions were compared. SUVs and ADC values were compared. AUROC analyses and cut-off values were obtained for each parameter. Mann–Whitney U, Spearman’s rho, and Kolmogorov–Smirnov tests were applied using SPSS. Results: The final study group included 58 bone lesions (19 patients (male: female = 6:13, age 52.5 ± 9.6, forty-four (75.9%) bone metastasis, fourteen (24.1%) benign red marrow hyperplasia). ADCs from BLADE and RESOLVE were significantly higher in bone metastasis than red marrow hyperplasia. BLADE showed higher ADC values, higher anatomical scores, and higher SNR than RESOLVE DWI (*p* < 0.05). Imaging times were longer for BLADE than RESOLVE (6 min 3 s vs. 3 min 47 s, *p* < 0.05). There was a poor correlation between ADC values and SUVs (correlation coefficient from 0.04 to 0.31). The AUROC values of BLADE and RESOLVE MRI ranged from 0.892~0.995. Those of PET ranged from 0.877~0.895. The cut-off ADC values between the bone metastasis and red marrow hyperplasia were 355.0, 686.5, 531.0 for BLADE min, max, and average, respectively, and 112.5, 737.0, 273.0 for RESOLVE min, max, and average, respectively. The cut-off SUV values were 1.84, 5.01, and 3.81 for mean, peak, and max values, respectively (*p* < 0.05). Conclusions: Compared with RESOLVE DWI, BLADE DWI showed improved image quality of pelvic bone MRI in the aspect of anatomical depiction and SNR, higher ADC values, albeit longer imaging time. BLADE and RESOLVE could differentiate bone metastasis and red marrow hyperplasia with quantifiable cut-off values. Further study is necessary to evaluate the discrepancy between the quantifiers between PET and MRI.

## 1. Introduction

Evaluating tumor response is essential in cancer treatment. Imaging biomarkers detect changes in the tumor, helping to determine therapy efficacy. For solid visceral tumors, criteria such as change in size or number, solid enhancement, sclerotic fill-in, the avidity of fluorine-18 fluorodeoxyglucose (18F-FDG) uptake have been incorporated into RECIST 1.1, PERCIST, and their variants. Yet, the criteria for bone metastasis, especially for lesions without extraosseous soft-tissue masses, are still limited [1].

Advanced imaging such as positron emission tomography (PET), PET/computed tomography (PET/CT), and magnetic resonance imaging (MRI) are important in the diagnosis and follow-up of malignant tumors. Yet, their application in quantifying bone metastases remains undefined. PET yields robust results on the molecular level, but its efficiency for musculoskeletal lesions is hampered due to nonspecific FDG uptake [2]. Magnetic resonance imaging (MRI), while it can offer valuable macromolecular information via diffusion-weighted imaging (DWI) and apparent diffusion coefficient (ADC), faces challenges that must be addressed to effectively integrate DWI sequences into the assessment of metastatic bone conditions. They include the cost, eddy current or susceptibility-induced artifacts, T2* blurring, and restricted spatial resolution, which becomes worse on 3T versus 1.5T scanners [3,4,5].

DWI techniques such as readout-segmented multi-shot echo planar imaging (RESOLVE) and turbo spin-echo-based radial TSE (BLADE) DWIs minimize some of the DWI drawbacks by reducing geometric distortion and artifacts [3,6,7,8] (K-space trajectory shown in Supplementary Figure S1). However, gaps exist in interpreting different DWI results and their association with PET-derived quantifiers. Furthermore, to the best of our knowledge, no studies have explored the efficacy of BLADE DWI for bone metastases.

The condition known as red marrow hyperplasia refers to the growth of hematopoietic cells replacing normal fatty (yellow) marrow in reaction to either a systemic demand or cellular hypofunction. Several conditions have been attributed, including chronic anemia, heavy smoking, and malnutrition, among others. The lesion frequently mimics bone metastasis on FDG PET and MRI without definitive diagnostic criteria [9,10].

Thus, this study’s goal is to compare BLADE DWI and RESOLVE DWI in imaging the pelvic bone metastasis correlate ADC with FDG uptake values in PET/CT. This study also seeks to compare the values of metastatic lesions with those of benign bone lesions, such as red marrow hyperplasia.

## 2. Materials and Methods

### 2.1. Patient Selection

**Figure 1:**
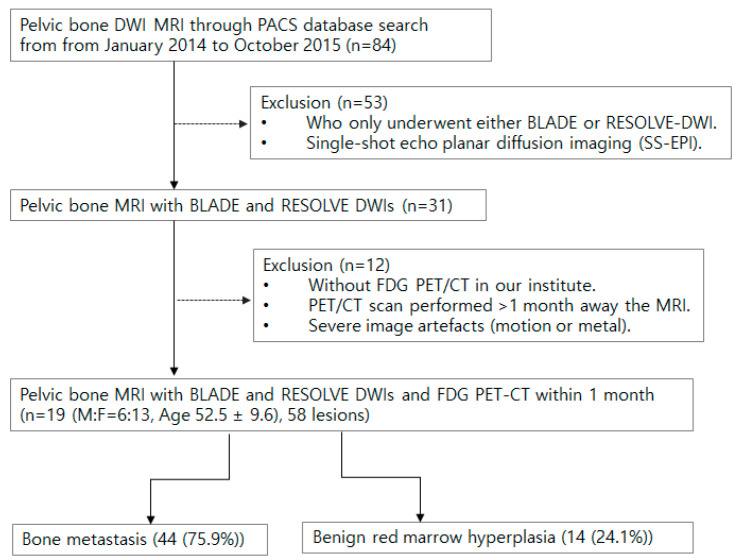
Flow chart for patient inclusion and exclusion.

This retrospective study from a single tertiary hospital involved 84 initial patients who underwent pelvic bone MRI with DWI between January 2014 and October 2015 by searching the Picture Archiving and Communication System (PACS). Exclusion criteria included those with only one type of DWI, having MRI and F-18 FDG PET/CT more than a month apart, and images with severe motion or metal artifacts (Figure 1). Among the eligible cases, bone metastasis and benign lesions were determined using best value comparator (BVS; a combination of imaging, clinical, and biological studies at the time of imaging, and at least 6 months of follow-up). More specifically, bone metastasis was defined as the combination of the following conditions: (1) bone marrow replacing lesion, which is low SI on T1WI, increased SI on FST2, and enhancement on postcontrast, (2) without visible intralesional fat component, (3) in patients with underlying malignancy. Red marrow was defined as; (1) nodular T1 low SI lesion in vertebrae or pelvic bones compared to adjacent fatty marrow, (2) low SI compared to marrow on T2WI and iso to slight high SI on FST2, and (3) no lesion progression for 6 months and in consensus of experts.

### 2.2. Magnetic Resonance Imaging

MRIs were performed with a 6-channel body coil at 3.0T (Verio; Siemens Healthcare GmbH, Erlangen, Germany), including precontrast T1, T2-weighted images, axial BLADE DWI (TR/TE 5200/104 ms, matrix 192 × 192, FOV 360 × 360 mm, slice thickness/gap 5/2.5 mm, B-value 50/800 s/mm^2^), and axial RESOLVE DWI (4900/62 ms, matrix 192/139, FOV 320/257 mm, slice thickness/gap 5/2.5 mm, B-value 50/800 s/mm^2^). Postcontrast T1-weighted images after the DWIs used the same parameters as pre-contrast images.

### 2.3. F-18 FDG PET/CT

Patients fasted 4–6 h before imaging and underwent scans if blood glucose levels were <200 mg/dL. 18F-FDG PET/CT scans were acquired 60 min post-injection of 185 MBq 18F-FDG, from skull base to upper thigh using a dedicated scanner (Biograph mCT 128; Siemens Healthcare GmbH, Erlangen, Germany). The final PET/CT images were analyzed on a dedicated workstation.

### 2.4. Quantification

Lesions with increased FDG uptake were identified on PET/CT by nuclear medicine physicians, blinded to the result of the MRI. Then, the MRI axial slice corresponding to these lesions was selected, and regions of interest (ROIs) were measured by the radiologist and nuclear medicine physician. The ovoid ROI was delineated to be as big as possible to fit within the bone marrow lesion. The measured SUV max, peak, and average, and ADC min, max, and average values were recorded for each lesion.

Image quality was assessed by two musculoskeletal radiologists, comparing the following structures on DWIs to postcontrast T1-weighted images: (1) visualization of SI joint, (2) visualization of sacral neural foramen, (3) the border between the tumor and surrounding bone marrow, (4) hamstrings at ischial tuberosity. We evaluated them according to a five-point Likert scale: structures unrecognizable (nondiagnostic 0), barely recognizable (poor 1), can be observed but vague than (fair 2), similar quality as (good 3), and better than (excellent 4), the fat-saturated postcontrast T1-weighted images.

The signal-to-noise ratio (SNR) of both DWIs was determined by regions of interest (ROIs) in the urinary bladder and the air regions outside the body and then calculated from the formula SNR = signal of ROI/(signal of air/0.66). Imaging times were documented.

### 2.5. Statistical Analysis

Average scores from two readers were used. The Mann–Whitney *U* test was used to compare ROI values and visual assessments. Spearman’s rho analyzed the correlation between MRI and PET values. Receiver operating characteristic (ROC) curves were generated, with cut-off values determined via the Kolmogorov–Smirnov (K-S) metric to differentiate metastatic from benign bone lesions. Statistical analysis used statistical software (SPSS, release 29.0, IBM, Armonk, NY, USA), and significance was set at *p* < 0.05.

#### Ethics

This retrospective study received approval from the institutional review board (IRB number GBIRB2023-055), and the requirement for informed consent was waived.

## 3. Results

The study group consisted of 44 (75.9%) bone metastases and 14 (24.1%) benign red marrow hyperplasia from 19 patients (M:F = 9:10, Age 52.5 ± 9.6). Cancers included ten breast, six lung, one esophageal, one rectal, and one liver origins.

The ADC values of metastatic lesions on both BLADE and RESOLVE DWIs were significantly higher than those of red marrow hyperplasia, as shown in Table 1. Similarly, SUVmax of metastatic bone lesions was higher than benign lesions (*p* < 0.05). The maximum and average ADC values from BLADE were significantly higher than those of RESOLVE DWI.

**Figure 2:**
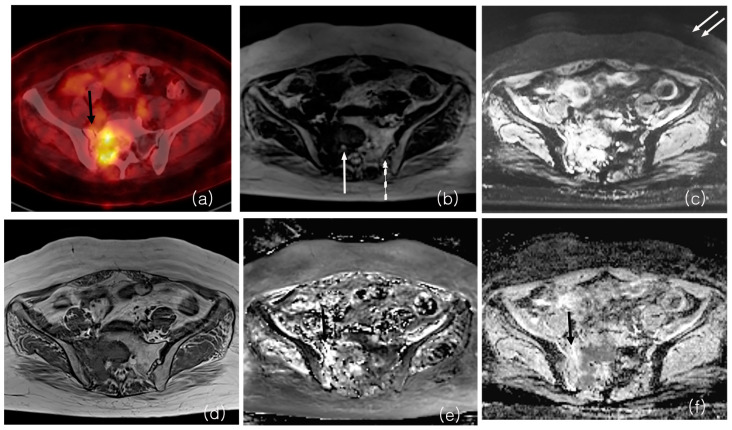
MRI and 18F-FDG PET/CT from a patient with bone metastasis from breast cancer. PET/CT shows a sacral lesion with increased FDG uptake, with SUV peak measuring 7.21 (**a**). BLADE b value 800 DWI (**b**) and ADC map (**e**) shows the lesion in the sacral body and right ala (ADC max 2924). RESOLVE b value 800 DWI (**c**) and ADC map (**f**) also shows the lesion (ADC max 2632). There is a fracture line in the right sacral ala, with less intense FDG uptake on PET and higher ADC value on MRI (black arrows, (**a**,**e**,**f**)). On image quality analysis, anatomical details such as neural foramen (white arrow) and sacroiliac joint (dotted arrow) are better visualized on BLADE (**b**) and less distinct on RESOLVE b value 800 DWI (**c**). Artifact in background air is more prominent on RESOLVE (double arrows on (**c**)). T1-weighted non-contrast MRI (**d**) is shown as a reference image.

**Figure 3:**
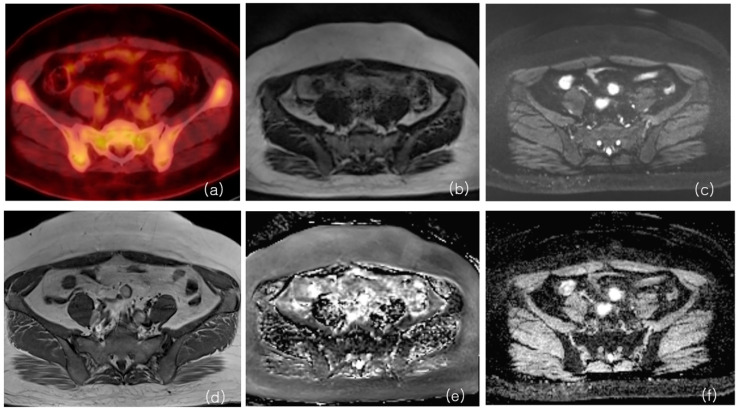
MRI and 18F-FDG PET/CT from a patient with red marrow hyperplasia. PET/CT shows a multifocal increased FDG uptake in bilateral iliac bone and sacrum (**a**). SUV peak from left iliac bone measured 3.15. The ADC max from BLADE b value was 800 DWI (**b**), and the ADC map (**e**) was 812. The ADC max from RESOLVE b value 800 DWI (**c**) and ADC map (**f**) was 454. T1-weighted non-contrast MRI (**d**) shows the bone marrow with diffuse intermediate SI, close to the signal intensity of muscle, compatible with red marrow.

Correlation coefficients of the ADC values from BLADE and RESOLVE DWI were 0.63 (Confidence interval (C.I.) 0.50~0.73), 0.61 (C.I. 0.47~0.72), and 0.73 (C.I. 0.63~0.81), for minimum, maximum, and average ADCs, respectively (*p* < 0.05), indicating strong correlation. The correlation between ADC values obtained using the BLADE or RESOLVE DWI methods and SUV was poor (ranging from 0.04 (C.I. −0.271~0.339, *p* = 0.810), to 0.31 (C.I. 0.01~0.57, *p* = 0.040). Correlation coefficients between DWI ADC and PET SUV are shown in Table 2, and representative images are shown in Figure 2 and Figure 3.

**Figure 4:**
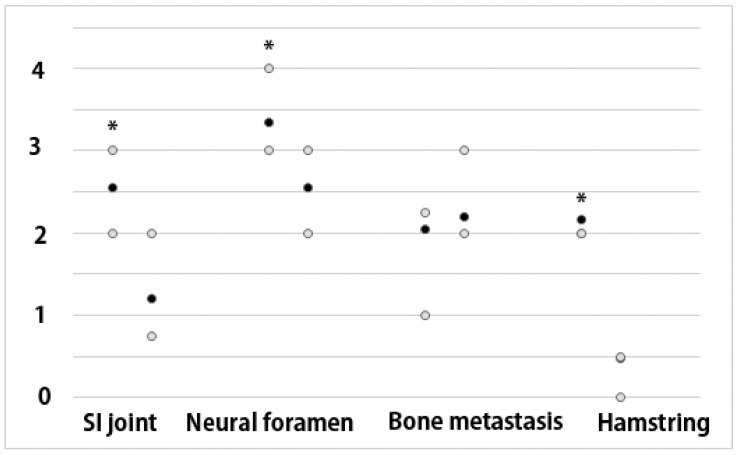
Anatomical Scores of BLADE and RESOLVE DWI MRI. (* Indicates values with *p* < 0.05). The average values are represented by the black dots, and the interquartile ranges by the gray dots. The scores of BLADE were significantly higher than RESOLVE DWI except for metastatic bone lesion visualization.

The anatomical scores of BLADE were significantly higher than RESOLVE DWI. The tumor border score was 2 in BLADE vs. 2.2 in RESOLVE, without a statistically significant difference (Figure 4).

The SNR and CNR of BLADE were significantly higher than those of RESOLVE (712.6 ± 236.5, vs. 216.4 ± 16.6, 193.5 ± 316.2, vs. 37.9 ± 28.5, respectively, *p* < 0.05). The imaging time of BLADE was significantly longer than RESOLVE (6 min 3 s ± 25 s vs. 3 min 47 s ± 16 s, respectively, *p* < 0.05). Table 3 summarizes the findings.

**Figure 5:**
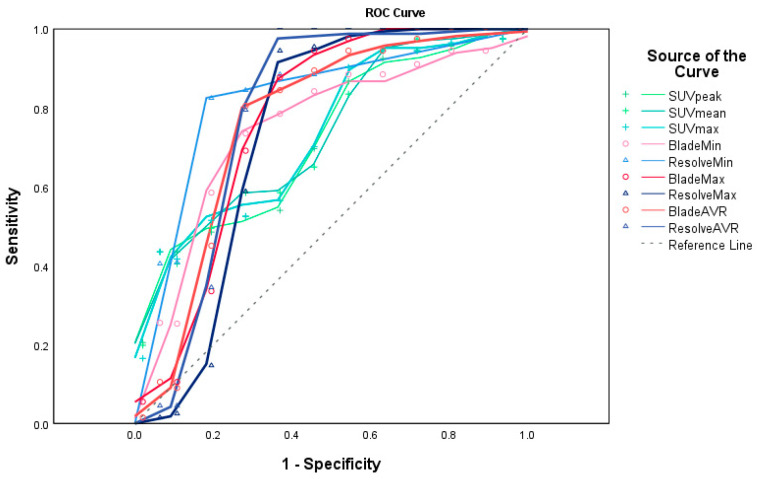
ROC curves for ADC of BLADE and RESOLVE MRI and SUV of 18F-FDG PET/CT (created by SPSS v29.0). The AUC values of BLADE (red lines and circles) and RESOLVE (blue lines and triangles) MRI ranged from 0.736~0.824. Those of PET (green lines and crosses) ranged from 0.732~0.745. (Reference line shown as a dashed gray line). The areas under the curve values are shown in Table 4.

Figure 5 and Table 4 show the AUROC curve values for ADC and SUV, as well as their cut-off values. The AUC values of BLADE and RESOLVE MRI ranged from 0.736~0.824. Those of PET ranged from 0.732~0.745. Estimated cut-off values of the malignant lesions were 423.5, 854.50, 720.50 for BLADE ADC min, ADC max, and ADC average, respectively, and 246.5, 737, 720.5 for RESOLVE ADC min, max, and ADC average, respectively. The cut-off values for PET were 2.1, 1.4, and 2.6 for peak, average, and max SUV values, respectively, with statistical significance.

## 4. Discussion

This work utilized BLADE and RESOLVE DWIs to image FDG-positive bone lesions, compared the diagnostic capacities, and assessed the correlation between their ADC and SUV. The BLADE DWI yielded higher ADC values, superior semiquantitative scores, and SNR compared to the RESOLVE, although with a longer scan time. There was minimal correlation between ADC and SUV in metastatic bone lesions. The BLADE and RESOLVE DWIs yielded ADC values, which were significantly higher in bone metastasis than benign red marrow hyperplasia.

Bone metastasis is a common occurrence in various types of solid cancers, with an incidence ranging from 50% to 80% in lung, prostate, and breast tumors [11]. Skeletal-related events (SREs) due to bone metastasis, such as pathologic fracture, hypercalcemia, or nerve compression, have a significant impact on the morbidity and prognosis of cancer patients. To prevent these occurrences, it is important to diagnose and characterize them early [12,13,14]. 18F-FDG PET/CT, a tool for simultaneous near-whole body screening of hypermetabolic lesions, is useful in metastasis localizations both in soft tissue and bone. It offers accurate and rapid whole-body images with high diagnostic accuracy and availability, and its diagnostic accuracy is higher than that of a bone scan. Nevertheless, there are several drawbacks associated with it, including the use of ionizing radiation and the difficulty in detecting minor lytic lesions, low glycolytic lesions, or cranial lesions. MRI has rapidly emerged as an important imaging tool to image malignancies and their associated complications. Quantitative MRI metrics, such as DWI ADC, provide numerical values that are important for evaluating many cancers. Malignant cells are highly cellular, and the DWI measurements from malignancy are known to demonstrate restricted diffusion. The ADC values are closely associated with the DWI acquisition parameters; in order for wider application in clinical practice, it is necessary to verify different DWI sequences against one another and compare them with traditional modalities such as PET. There have been only a few studies in the literature that have compared two different MRIs and PET/CT simultaneously for evaluating bone metastasis and benign red marrow hyperplasia [15,16].

One of the key findings of this study was that BLADE DWI, based on a fast spin echo sequence, improved visualization of pelvic lesions with increased SNR, suggesting that it is potentially better for complex musculoskeletal structures. The BLADE acquires the data in radial trajectory and is a variant of a PROPELLER (Periodically Rotated Overlapping ParalEL Lines with Enhanced Reconstruction) (Supplement Figure S1). Due to the lack of unique frequency or phase encoding direction, noise from moving anatomy does not propagate as an artifact along a single direction and oversampling in the center of K space in all directions. The technique has been employed in the pediatric population, as well as for imaging of the head and abdomen, and has exhibited improved image quality in patients with voluntary or involuntary movement [17,18,19]. The BLADE technique in our institute was employed in abdominopelvic imaging to address the issue of artifacts caused by pulsation and susceptibility, which present a considerable obstacle when imaging pelvic bone metastases. We were able to demonstrate that the BLADE indeed reduced anatomical distortion of curvilinear structures, such as the sacroiliac joint, margin of neural foramina, and hamstring attachment sites, compared to the RESOLVE technique. Additionally, the BLADE images exhibited fewer artifacts in the air surrounding the body, and SNR also increased with the approach. Nevertheless, it is crucial to consider the extended scan time, and it is advisable to use a single consistent DWI technique for follow-up examinations of cancer patients rather than abruptly switching between DWI sequences, given the substantial disparity in apparent diffusion coefficient (ADC) values between BLADE and RESOLVE.

The second finding was that while both SUV and ADC offered thresholds to differentiate between bone metastasis and red marrow hyperplasia, no strong correlation was observed between these measures. DWI is a technique that relies on the random movement of water molecules, known as Brownian motion. It has been observed that there is an inverse relationship between the apparent diffusion coefficient (ADC) and cellularity. Consequently, malignant lesions tend to exhibit lower ADC values compared to benign lesions, while an increase in ADC is observed in lesions with treatment response [20]. Previous literature had varied correlation findings between PET and DWI for nonosseous malignancy [21,22,23,24,25]. Neither of the methods is cancer cell-specific and depends on various factors such as tracer metabolism and tissue composition [26]. Benign bone lesions such as red marrow hyperplasia, indolent neoplasm, inflammation, or fracture can also show high FDG uptake, mimicking metastasis. On the other hand, aggressive metastatic lesions may show low FDG uptake, and they may not be detectable with the naked eye [27]. Moreover, the difference in FDG uptake exists between osteolytic metastatic lesions and osteosclerotic ones [28,29]. Although both the DWI and PET have improved the diagnosis of malignant bone lesions, each of them needs thorough assessment for consistency and interchangeability before regular clinical use.

Red marrow hyperplasia can be attributed to various factors, such as the administration of marrow-stimulating drugs and persistent physiologic stress, hence affecting a considerable number of cancer patients. The imaging characteristics of this condition include increased FDG uptake and loss of fat signal intensity on T1-weighted MRI [10,30,31]. Its appearance is challenging to diagnose in cancer patients. As far as we know, DWI has not been rigorously applied to compare the condition with bone metastasis.

It is noteworthy that malignant bone lesions had higher ADC values than red marrow hyperplasia. The ADC demonstrates an inverse relationship with cellularity. However, it is possible for a decrease in Brownian motion to take place in lesions characterized by fibrosis and trabecular structures lacking edema, such as normal background bone marrow. This phenomenon is observed regardless of the level of cellularity. The lower ADC value observed in the red marrow compared to bone metastases can be attributed to the presence of fat and trabecular bone within the red marrow. The cut-off values to diagnose bone metastasis would, therefore, be higher than those for red marrow hyperplasia.

There are several limitations in this study. First, it was conducted retrospectively at a single institution, introducing bias in the selection of research participants. Secondly, although histopathological examination is the gold standard for the diagnosis of bone lesions, tissue samples were not obtained for practical reasons such as advanced disease. Thirdly, there being diverse types of primary cancer, the nature of bone metastases may differ depending on the primary cancer. Lastly, the limited availability of all three modality-acquisition for cancer patients resulted in a small number of patients. Future research will be conducted with a larger patient group with power calculation to assess the effectiveness of BLADE with various scanners and parameters.

## 5. Conclusions

The BLADE MRI outperformed RESOLVE in imaging pelvic bone metastasis in terms of anatomical depiction, SNR, and ADC values. BLADE may be a more effective DWI sequence for cases requiring good anatomical depiction. The observed disparity of metrics and the weak connection between PET and MRI necessitate further research.

## Figures and Tables

**Table 1:** Quantifiable variables from MRI and PET. ADC minimum (min), maximum (max), and average (avr) values from BLADE and RESOLVE DWIs, SUV peak, mean, and maximum from F-18 FDG PET for bone metastasis and benign red marrow hyperplasia are listed below. Asterisks indicate the values showing statistically significant differences between malignant and benign lesions.

Modality	Metrics	Metastasis	Red Marrow
BLADE	ADCMin	600.9 ± 57.9 (*p* = 0.01) *	136 ± 61.7
	ADCMax	1460.4 ± 107.8 (*p* = 0.002) *	413.4 ± 74.4
	ADCAvr	972.8 ± 71.6 (*p* = 0.002) *	260.2 ± 71.9
RESOLVE	ADCMin	610.1 ± 67.3 (*p* = 0.005) *	22.0 ± 22.0
	ADCMax	1250.4 ± 82.2 (*p* = 0.005) *	523.8 ± 81.8
	ADCAvr	883.9 ± 8.2 (*p* = 0.002) *	166.2 ± 34.4
F-18 FDG PET	SUVpeak	4.9 ± 0.5 (*p* = 0.03) *	1.6 ± 0.1
	SUVmean	3.3 ± 0.2 (*p* = 0.006) *	1.4 ± 0.2
	SUVmax	5.4 ± 0.5 (*p* = 0.018) *	1.9 ± 0.2

**Table 2:** Correlation coefficient between ADC values from BLADE and RESOLVE and SUV values from PET (* indicates the value with statistical significance (*p* < 0.05)). The only statistically significant correlation was noted between maximum ADC (ADCMax) from RESOLVE and SUVpeak, with a correlation coefficient of 0.31; there was a poor correlation between ADC and SUV (C.I. denotes confidence interval).

R^2^		SUVpeak	C.I., *p* Values	SUVmean	C.I., *p* Values	SUVmax	C.I., *p* Values
BLADE	ADCMin	0.1	(−0.21~0.39, *p* = 0.510)	0.04	(−0.271~0.339, *p* = 0.810)	0.07	(−0.239~0.368, *p* = 0.650)
	ADCMax	0.21	(−0.10~0.48, *p* = 0.170)	0.14	(−0.177~0.423, *p* = 0.380)	0.14	(−0.175~0.425, *p* = 0.370)
	ADCAvr	0.23	(−0.08~0.50, *p* = 0.130)	0.16	(−0.155~0.441, *p* = 0.310)	0.16	(−0.156~0.440, *p* = 0.310)
RESOLVE	ADCMin	0.11	(−0.20~0.40, *p* = 0.470)	0.07	(−0.239~0.369, *p* = 0.640)	0.1	(−0.216~0.390, *p* = 0.540)
	ADCMax	0.31 *	(0.01~0.57, *p* = 0.040)	0.27	(−0.038~0.532, *p* = 0.070)	0.24	(−0.074~0.505, *p* = 0.120)
	ADCAvr	0.23	(−0.08~04, *p* = 0.130)	0.19	(−0.124~0.466, *p* = 0.220)	0.2	(−0.117~0.472, *p* = 0.200)

**Table 3:** Signal-to-noise ratio (SNR) and imaging times of BLADE DWI and RESOLVE DWI. The values are significantly higher (*) in BLADE compared with RESOLVE DWI.

	BLADE DWI	RESOLVE DWI
SNR	712.6 ± 236.5 *	216.4 ± 16.6
Imaging Time	6 min 3 s ± 25 s *	3 min 47 s ± 16 s

**Table 4:** Area Under the ROC Curve. The AUC values of BLADE and RESOLVE MRI ranged from 0.736~0.824. Those of PET ranged from 0.732~0.745. The cut-off values between the malignant and benign lesions were 423.5, 854.50, 720.50 for BLADE ADC min, ADC max, and ADC average, respectively, and 246.5 m 737, 720.5 for RESOLVE ADC min, max, and ADC average, respectively. The cut-off values for PET were 1.84, 5.01, and 3.81 for minimum, peak, and mean SUV values, respectively (*p* < 0.05).

Variables		Area	Cut-Off Value	Sensitivity (%)	Specificity (%)
BLADE	ADC Min	0.891	355.0	77.3	100
	ADC Max	0.982	686.5	90.9	100
	ADC Average	0.950	531.0	818	100
RESOLVE	ADC Min	0.918	112.50	81.8	100
	ADC Max	0.982	737.0	90.9	100
	ADC Average	0.995	273.0	97.7	100
FDG-PET	SUV peak	0.877	2.06	79.5	100
	SUV mean	0.889	1.44	88.6	80
	SUV max	0.895	2.59	77.3	100

## Data Availability

The datasets generated during and/or analyzed during the current study are not publicly available because they are medical data from patients but are available from the corresponding author on reasonable request.

## Supplementary Materials

The following supporting information can be downloaded at: https://www.mdpi.com/article/10.3390/cancers16010214/s1, Figure S1: K-space trajectory of RESOLVE DWI and BLADE DWI.
