# Supplementary material for: Evaluation of Three Imaging Methods to Quantify Key Events in Pelvic Bone Metastasis
Source: Cancers (Basel). 2024 Jan 2;16(1):214. doi: 10.3390/cancers16010214 (PMC10778360; doi:10.3390/cancers16010214)
Supplement: Supplementary file 1 [file cancers-16-00214-s001.zip › cancers-2746453-supplementary.pdf]

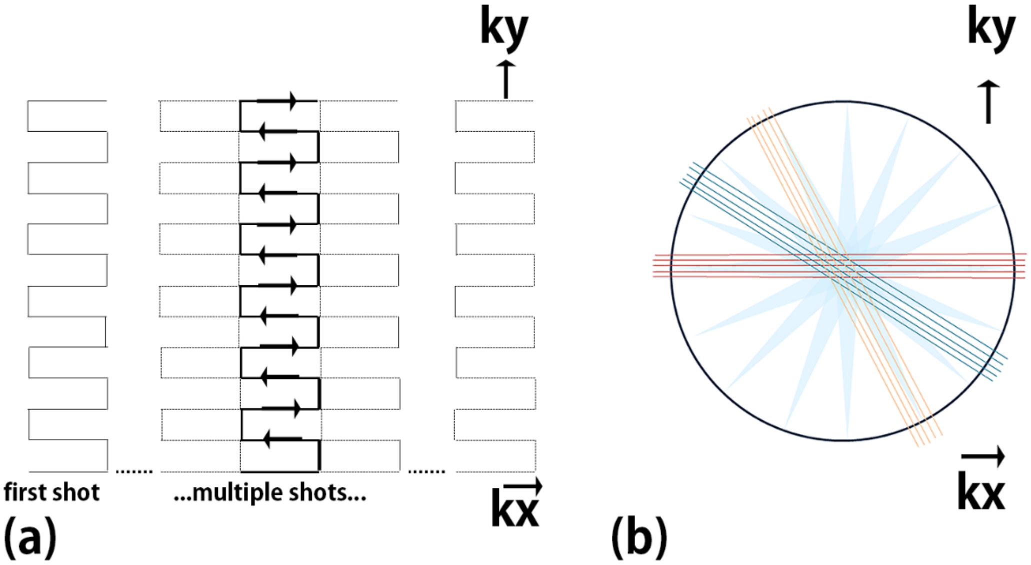

Figure S1. K-space trajectory of RESOLVE DWI and BLADE DWI. In RESOLVE DWI, the k-space trajectory is divided into multiple shots in the readout direction (kx), which allows for an encoding-time reduction increasing image quality. BLADE acquires every *k*-space line at the center of a spin echo, thus avoiding the phase accumulation that leads to distortion and blurring in EPI. The center of the *k*-space is covered in each train of refocusing pulses, and multiple rotated blades are acquired to fully sample a circular *k*-space region over multiple shot.
